# Transcriptional regulatory networks underlying the reprogramming of spermatogonial stem cells to multipotent stem cells

**DOI:** 10.1038/emm.2017.2

**Published:** 2017-04-14

**Authors:** Hoe-Su Jeong, Jinhyuk Bhin, Hyung Joon Kim, Daehee Hwang, Dong Ryul Lee, Kye-Seong Kim

**Affiliations:** 1 Hanyang University College of Medicine, Graduate School of Biomedical Science and Engineering, Seoul, Republic of Korea; 2 Department of Chemical Engineering, POSTECH, Pohang, Republic of Korea; 3 Department of New Biology and Center for Plant Aging Research, Institute for Basic Science, Daegu Gyeongbuk Institute of Science and Technology, Daegu, Republic of Korea; 4 CHA Stem Cell Institute, CHA University, Seoul, Republic of Korea

## Abstract

Spermatogonial stem cells (SSCs) are germline stem cells located along the basement membrane of seminiferous tubules in testes. Recently, SSCs were shown to be reprogrammed into multipotent SSCs (mSSCs). However, both the key factors and biological networks underlying this reprogramming remain elusive. Here, we present transcriptional regulatory networks (TRNs) that control cellular processes related to the SSC-to-mSSC reprogramming. Previously, we established intermediate SSCs (iSSCs) undergoing the transition to mSSCs and generated gene expression profiles of SSCs, iSSCs and mSSCs. By comparing these profiles, we identified 2643 genes that were up-regulated during the reprogramming process and 15 key transcription factors (TFs) that regulate these genes. Using the TF-target relationships, we developed TRNs describing how these TFs regulate three pluripotency-related processes (cell proliferation, stem cell maintenance and epigenetic regulation) during the reprogramming. The TRNs showed that 4 of the 15 TFs (*Oct4/Pou5f1*, *Cux1*, *Zfp143* and *E2f4*) regulated cell proliferation during the early stages of reprogramming, whereas 11 TFs (*Oct4/Pou5f1*, *Foxm1*, *Cux1*, *Zfp143*, *Trp53*, *E2f4*, *Esrrb*, *Nfyb*, *Nanog*, *Sox2* and *Klf4*) regulated the three pluripotency-related processes during the late stages of reprogramming. Our TRNs provide a model for the temporally coordinated transcriptional regulation of pluripotency-related processes during the SSC-to-mSSC reprogramming, which can be further tested in detailed functional studies.

## Introduction

Spermatogonial stem cells (SSCs) are the germline stem cells located along the basement membrane of seminiferous tubules in mammalian testes. SSCs are self-renewing and undifferentiated cells in the testicular microenvironment. In response to differentiation cues, they can be differentiated into one specialized cell lineage, spermatozoa. Recent studies have shown that these unipotent SSCs can be reprogrammed into multipotent SSCs (mSSCs) under defined culture conditions.^1, 2, 3, 4, 5, 6^ Unlike embryonic stem cells (ESCs) and induced pluripotent stem cells (iPSCs) that have several issues, such as tumorigenic potential and ethical concerns, hindering their application in regenerative medicine, these mSSCs can serve as a pluripotent stem cell source free from these issues.

Previous studies have focused on both the generation and characterization of mSSCs. Comparative analyses of gene expression profiles revealed that mSSCs were similar to ESCs, compared with iPSCs, SSCs and neural stem cells (NSCs).^6, 7^ In addition, mSSCs were also similar to ESCs in their miRNA expression profiles. For example, mSSCs highly express a set of ESC-specific miRNAs, including the miR-290 and miR-302 families.^8^ Moreover, a comparative analysis of proteome profiles demonstrated that mSSCs had a highly similar proteome to that of ESCs. Furthermore, DNA methylation profiles, as well as an analysis of the methylation of pluripotency marker gene loci, showed significant similarities between mSSCs and ESCs.^9^ These similarities between mSSCs and ESCs at multiple molecular levels strongly support the idea that mSSCs acquire naïve pluripotent states similar to those of ESCs.^10^

Transcriptional regulatory networks (TRNs) are essential to understanding the reprogramming of SSCs to mSSCs. Pluripotency-inducing transcription factors (TFs), such as Oct4/Pou5f1, Sox2, Myc and/or Klf4, have key roles in the reprogramming and generation of iPSCs.^11, 12^ Several studies have also reported a few other factors that influence the reprogramming of SSCs to mSSCs. For example, SSCs derived from p53-knock-out mice can be converted into ES-like cells more quickly than those derived from wild-type mice, implying that p53 negatively influences the reprogramming process.^5^ The microRNAs that regulate the levels of mRNAs involved in SSC reprogramming, such as *miR-372*, *miR-373* and *miR-470*, can act as potential regulators of the reprogramming.^13^ Despite all these findings, the TRNs underlying SSC reprogramming remain unclear.

Here, we present a systems approach used to identify novel TFs that are important for reprogramming and to decode a TRN underlying the reprogramming, which describes how the novel and known TFs control cellular processes related to the reprogramming of SSCs to mSSCs. We first generated intermediate SSCs (iSSCs) undergoing the transition to mSSCs and then performed gene expression profiling of SSCs, iSSCs and mSSCs. Comparisons of the gene expression profiles revealed the reprogramming-related genes that increased in expression during the course of the reprogramming of SSCs to mSSCs. By identifying key TFs that regulate the reprogramming-related genes, we developed a TRN that describes the regulation of three reprogramming-related processes (cell proliferation, aerobic glycolysis and epigenetic regulation) by these TFs, which could improve the understanding of the reprogramming of SSCs to mSSCs.

## Materials and methods

### Microarray data analysis

Total RNA was isolated from SSCs, iSSCs and mSSCs using an RNeasy mini kit (Qiagen, Valencia, CA, USA), and RNA integrity was assessed using a Bioanalyzer 2100 instrument (Agilent, Santa Clara, CA, USA). The RNA integrity numbers (RINs) were higher than 8 for all samples. Biological replicates were obtained from independently cultured SSCs (*n*=3), mSSCs (*n*=3) and iSSCs (*n*=2). For all replicates, the total RNA was reversed-transcribed into cDNA, amplified and then hybridized onto an Illumina MouseRef-8-v2-BeadChip gene expression array, according to the standard protocols of the manufacturer. The array was then scanned using the BeadStation 500 System (Illumina, San Diego, CA, USA) to quantify the signal of the probes. We normalized the log_2_ intensities of all probes using the quantile normalization method.^14^ The raw data were deposited into the gene expression omnibus (GEO) database (Accession ID: GSE86072).

### Identification of differentially expressed genes

Before identifying the differentially expressed genes (DEGs), we first defined the expressed genes across SSCs, iSSCs and mSSCs using a Gaussian mixture modeling method as previously described.^15^ Among the expressed genes, we identified the DEGs from the following three comparisons: (1) SSCs versus iSSCs, (2) iSSCs versus mSSCs and (3) SSCs versus mSSCs, using the previously reported integrative statistical hypothesis testing method^16^ that computes adjusted *P*-values (*P*) by combining a two-sample *t*-test and the median ratio test. For each comparison, the DEGs were selected from the expressed genes as the ones with *P*<0.05 and log_2_ fold changes >0.58. A total of 6627 DEGs were identified from the three comparisons.

### Categorization of DEGs by temporal expression patterns

We categorized the 6627 DEGs into 18 clusters based on their differential expression patterns in the three comparisons (SSCs versus iSSCs, iSSCs versus mSSCs and SSCs versus mSSCs) (Supplementary Figure S4a). The statistical testing method employs the discrete decision of whether a gene shows a statistically significant change between two conditions, and this discrete decision cannot detect marginal expression changes of the gene. To incorporate the marginal changes into the clustering of DEGs, we further performed a second clustering that clusters the mean profiles of the 18 clusters (Supplementary Figure S4b) using a hierarchical clustering method (Euclidean distance and average linkage). From the hierarchical clustering result, we finally identified twelve groups of DEGs (groups 1–12) such that the DEGs in each group showed the same differential expression pattern (Supplementary Figure S4c).

### Identification of the cellular processes enriched by the genes in groups 1–12

To identify the cellular processes associated with the DEGs in groups 1–12, we performed an enrichment analysis of GOBPs for the DEGs in each group using DAVID Bioinformatics Resources V6.7.^17^ The GOBPs with a *P*-value <0.1 (the default cut off in DAVID) and a count ⩾3 were selected as cellular processes enriched by the genes in each group.

### Identification of major TFs

To identify major TFs for a set of DEGs, we performed a TF target enrichment analysis for the DEGs in each of groups 1–12 using MetaCore,^18^ which calculated the significance (*P*-value) of the number of targets of each TF from a hypergeometric distribution. For the multiple hypothesis testing correction, MetaCore computed a false discovery rate (FDR) from the *P*-value for each TF. The TFs with an FDR <0.05 and a target gene count ⩾5 were defined as major TFs in each group. Some TFs, including *Creb1*, *Sp1* and *Sp2*, were excluded from the major TFs because they had significant numbers of non-specific targets in almost all of the groups.

### Identification of pluripotency-related groups of DEGs

To examine which groups of DEGs showed significant overlaps with pluripotency-related genes in mESCs, we used the previously reported^19^ gene expression profiles (GSE2972, GSE3749 and GSE3231) generated from three mESC lines with different genetic backgrounds, R1, V6.5 and J1, and EBs after differentiation (6, 12, 18, 24 and 36 h, and 2, 4, 7, 9 and 14 days). The log_2_ intensities were normalized using the GC-RMA package in Bioconductor.^20^ In each time-course dataset, log_2_ fold changes for each probe set were then calculated by subtracting the log_2_ intensity of the mESCs from those at all time points, resulting in a *k* × 10 log_2_ fold-change matrix, where *k* is the number of probe sets. In total, three fold-change matrices were generated. After transforming each of these three matrices into a single vector, the three vectors were normalized using the quantile normalization method to avoid bias toward datasets with large fold-changes and then were transformed back into three matrices. Finally, the three matrices with normalized log_2_ fold changes were combined by matching the probe set IDs, resulting in a combined log_2_ fold-change matrix. Non-negative matrix factorization (NMF) was then applied to the combined matrix as previously described.^21^ The number of clusters was set to be 40. The genes belonging to each cluster were selected as the ones with a *P*-value <0.05. From the 40 clusters, the clusters showing an overall up- and down-regulation during differentiation across all three mESC lines were selected as differentiation- and pluripotency-related genes (with a fold change ⩾1.5 for at least one time point during differentiation), respectively. The *P*-value for the overlap of the differentiation- or pluripotency-related genes with each group of DEGs was computed based on the null hypothesis distribution of the number of overlapped genes using random sampling experiments for the DEG group being analyzed.

### Reconstruction of the TRNs delineating cell proliferation, glycolysis and epigenetic regulation

To reconstruct TRNs for each of three pluripotency-related processes (stem cell maintenance, cell proliferation-related processes and epigenetic regulation), we first selected the DE-mTFs that strongly regulate the genes involved in the process (Figure 4d; see ‘Results' section) and then targeted the genes in group 1 or 3 with the GOBPs for the process based on the protein–DNA interactions from MetaCore.^18^ Using the sets of TFs and target genes and the relationships between the TFs and target genes, the TRN for each of the three processes was then reconstructed and displayed using Cystoscope software.^22^ Protein–protein interactions among the nodes (TFs and target genes) were obtained from the following interactome databases and then added to the TRN: BioGRID,^23^ IntAct,^24^ MINT,^25^ DIP^26^ and I2D.^27^ The nodes in the TRN were arranged based on the KEGG pathway associated with each of the three pluripotency-related processes.

### Reverse transcription-polymerase chain reaction

For the reverse transcription-polymerase chain reaction (RT-PCR) analysis, 1 μg of DNase I-treated total RNA was reverse-transcribed using Superscript II reverse transcriptase with random hexamers (Invitrogen, Carlsbad, CA, USA) according to the manufacturer's instructions. The standard PCR conditions were as follows: 4 min at 94 °C, followed by 25–35 cycles of 30 s at 94 °C, 30 s at 55 °C and 30 s at 72 °C. The primer sequences used in this study are given in Supplementary Table S6.

## Results

### Gene expression signatures related to the reprogramming of SSCs to mSSCs

**Figure 1:**
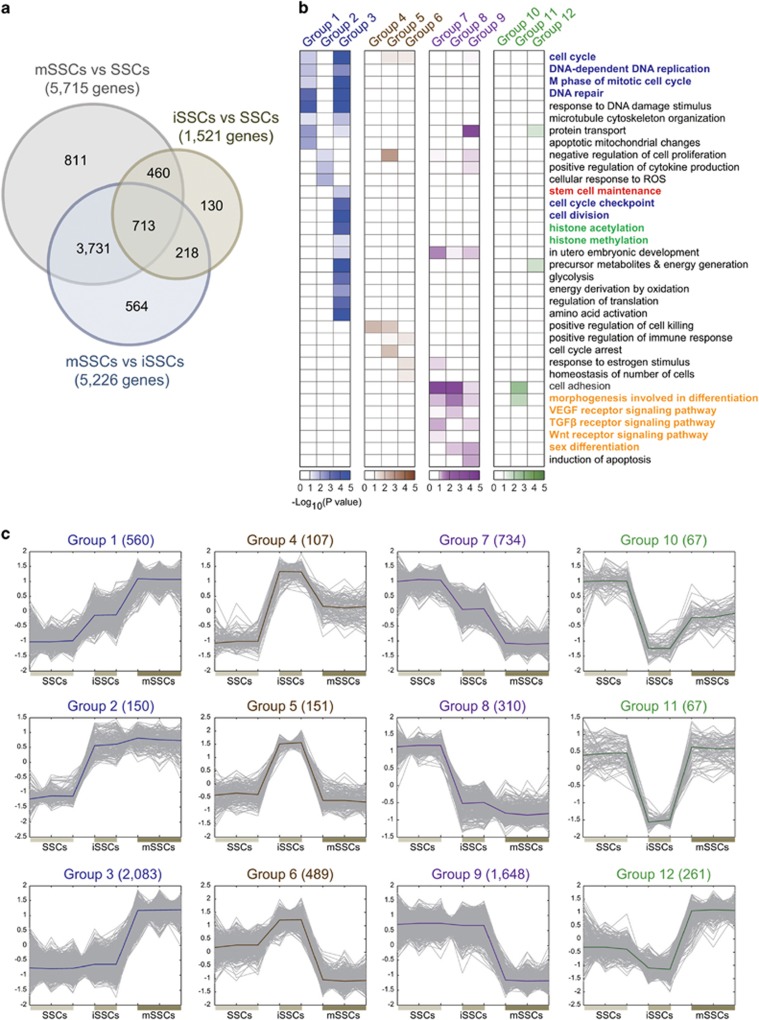
DEGs during the reprogramming of SSCs to mSSCs. (**a**) DEGs identified from the three comparisons (mSSCs versus SSCs, mSSCs versus iSSCs and iSSCs versus SSCs). The Venn diagram shows the relationships among the DEGs in individual comparisons. The numbers in parentheses denote the numbers of DEGs identified in the individual comparisons. (**b**) Different expression patterns of DEGs in groups 1–12 during the reprogramming of SSCs to mSSCs. To clearly show the temporal differential expression patterns, the gene expression data in individual groups of DEGs were normalized to have zero mean values and unit standard deviations. Thick lines represent the median profiles of gene expression in groups 1–12. *X*-axis, replicates in SSCs, iSSCs and mSSCs; Y-axis, auto-scaled expression levels. (**c**) GOBPs associated with the genes in groups 1–12. The color in the heat maps represents −log_10_(*P*), in which *P* is the enrichment *P*-value obtained from the DAVID software for the corresponding GOBP. Different colors were used for four different groups of DEGs. In addition, the colored GOBP labels indicate the three pluripotency-related processes (stem cell maintenance (red), cell proliferation-related processes (blue), and epigenetic regulation (green)) and differentiation-related pathways and processes (orange).

To understand the reprogramming of SSCs to mSSCs, we first developed mSSCs derived from SSCs as previously described.^28^ During the SSC-to-mSSC reprogramming, colonies of iSSCs appeared after two to three passages, and we then isolated and grew these iSSCs in the ESC culture medium for nine additional passages, leading to mSSCs. To identify the gene expression signatures related to the SSC-to-mSSC reprogramming, we first performed gene expression profiling of SSCs, iSSCs and mSSCs using an Illumina MouseRef-8 v2.0 BeadChip containing probes for 17 860 annotated genes. Using the resulting gene expression profiles, we then performed the following comparisons: (1) iSSCs versus SSCs, (2) mSSCs versus SSCs and (3) mSSCs versus iSSCs. From these three comparisons, we identified 1521, 5715 and 5226 DEGs, respectively (Figure 1a). To understand the temporal transition of gene expression during the SSC-to-mSSC reprogramming, we next categorized the DEGs into 12 groups (Figure 1b) based on their differential expression patterns in the three comparisons as described in ‘Materials and Methods' section (Supplementary Table S1). Of these groups, groups 1 and 7 (560 and 734 DEGs, respectively) showed a gradual increase and decrease in expression, respectively, during reprogramming. Groups 2 and 8 (150 and 310 DEGs, respectively) showed an increase and decrease, respectively, during the early transition from SSCs to iSSCs and then maintained these changes during the late transition from iSSCs to mSSCs, whereas groups 3 and 9 (2083 and 1648 DEGs, respectively) showed expression changes specifically during the late transition. Finally, groups 4–6 and 10–12 showed expression changes during the early transition, but restored the changes partially (groups 4 and 10), completely (groups 5 and 11) or excessively (groups 6 and 12) during the late transition. These data indicate that the SSC-to-mSSC reprogramming involves various modes of temporal gene expression changes.

### Cellular processes associated with the reprogramming of SSCs to mSSCs

To examine the cellular processes associated with the SSC-to-mSSC reprogramming, we then identified the Gene Ontology Biological Processes (GOBPs) represented by the twelve groups of DEGs using the DAVID software^17^ (Figure 1c; Supplementary Table S2). Of the twelve groups, group 3, showing late changes during the reprogramming, was associated with cellular processes related to pluripotency and self-renewal, including (1) stem cell maintenance, (2) cell proliferation-related processes (cell cycle, DNA replication, M phase of mitotic cell cycle, DNA repair, cell cycle checkpoint and cell division) and (3) epigenetic regulation-related processes (histone acetylation and methylation). Of these processes, several of the cell proliferation-related processes (cell cycle, DNA replication, M phase of mitotic cell cycle and DNA repair) were commonly associated with group 1, which showed early changes during the reprogramming. These data suggest that cell proliferation began to be activated in the early transition from SSCs to iSSCs (group 1), whereas the other pluripotency-related processes were activated primarily during the late transition from iSSCs to mSSCs (group 3). Moreover, interestingly, groups 7–9, showing early or late decreases in expression, were associated with cellular processes (morphogenesis for differentiation and sex differentiation) and signaling pathways (*TGFβ*, *Wnt* and *VEGF* signaling pathways)^29^ related to the differentiation of SSCs, suggesting that these differentiation-related processes are inactivated during the reprogramming. Thus, these data collectively indicate that the SSC-to-mSSC reprogramming involves a transcriptional program that involves the temporally coordinated activation and inactivation of pluripotency- and differentiation-related processes during the course of the reprogramming.

### Identification of the major TFs associated with the SSC reprogramming

**Figure 2:**
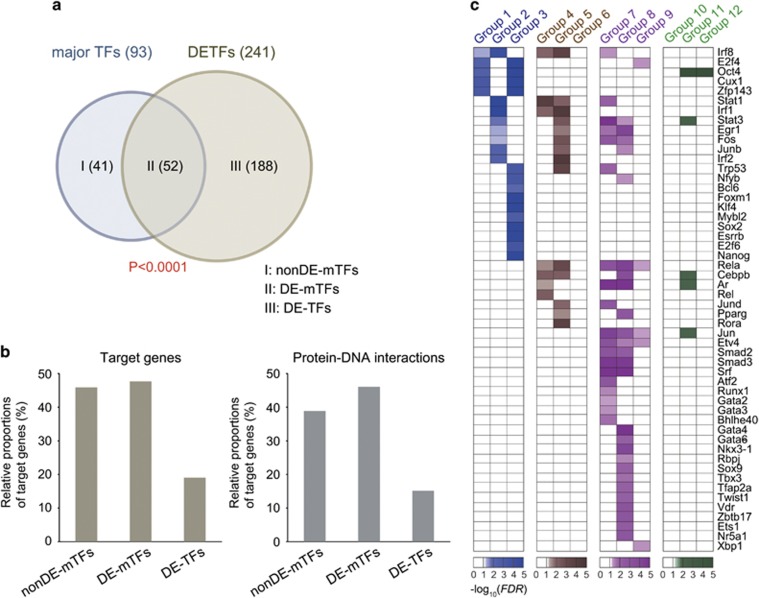
Major TFs predominantly regulating the DEGs during the reprogramming of SSCs to mSSCs. (**a**) Venn diagram showing the relationships among 93 major TFs and 241 DETFs. These TFs were categorized into three groups (I: 41 non-DE-mTFs; II: 52 DE-mTFs; and III: 188 DE-TFs). The overlap between the two sets of TFs was found to be significant (*P*<1 × 10^−4^ from hypergeometric test). (**b**) Relative proportions of DEGs regulated by the three groups of TFs (target genes, left) and the numbers of links between DEGs and the three groups of TFs (Protein–DNA interactions, right). (**c**) Heat maps showing the significance of the target genes in groups 1–12 that are regulated by the 52 DE-mTFs. The FDR was computed as the significance measure (‘Materials and Methods' section). The color represents –log_10_(FDR). Different colors were used for four different groups of DEGs.

Transcriptional regulation is a primary mechanism necessary to acquire pluripotency, as demonstrated in the reprogramming of human and mouse fibroblasts to iPSCs. Pluripotency-inducing TFs, such as *Oct4/Pou5f1*, *Sox2*, *Myc* and *Klf4*, have been used to reprogram fibroblasts into iPSCs.^11, 12^ Thus, decoding the TRNs underlying the gene expression changes in groups 1–12 (Figure 1b) is essential to understanding the SSC-to-mSSC reprogramming. To decode the core transcriptional regulations, we first identified 93 major TFs that had significant (FDR<0.05 and number of target genes ⩾5) numbers of target genes in groups 1–12 based on the TF-target gene information obtained from MetaCore.^18^ Moreover, the differentially expressed TFs (DETFs) during the course of the reprogramming could significantly contribute to transcriptional regulation of their target genes in groups 1–12. Thus, we further identified 241 DETFs among the genes in groups 1–12. These two sets of major TFs and DETFs can be categorized into three groups (Figure 2a): (1) 41 non-differentially expressed major TFs (non-DE-mTFs), (2) 52 differentially expressed major TFs (DE-mTFs) and (3) 188 differentially expressed, but non-major TFs (DE-TFs). We then examined the relative proportions of the DEGs that can be regulated by the three TF groups. Of the three groups, the 52 DE-mTFs had the largest numbers of target genes in groups 1–12 and links to the target genes (Figure 2b, left and right, respectively; Supplementary Table S3). Moreover, the 52 DE-mTFs had significant numbers of target genes across all the groups, except for group 10, which was the smallest among groups 1–12 (Figure 2c). These data indicate that of the three groups of TFs, the 52 DE-mTFs contribute most significantly to the transcriptional regulation of the DEGs in groups 1–12. Thus, we focused on these 52 DE-mTFs for the analysis of the TRNs underlying the SSC-to-mSSC reprogramming.

### Transcriptional regulatory relationships between major TFs and pluripotency-related genes

**Figure 3:**
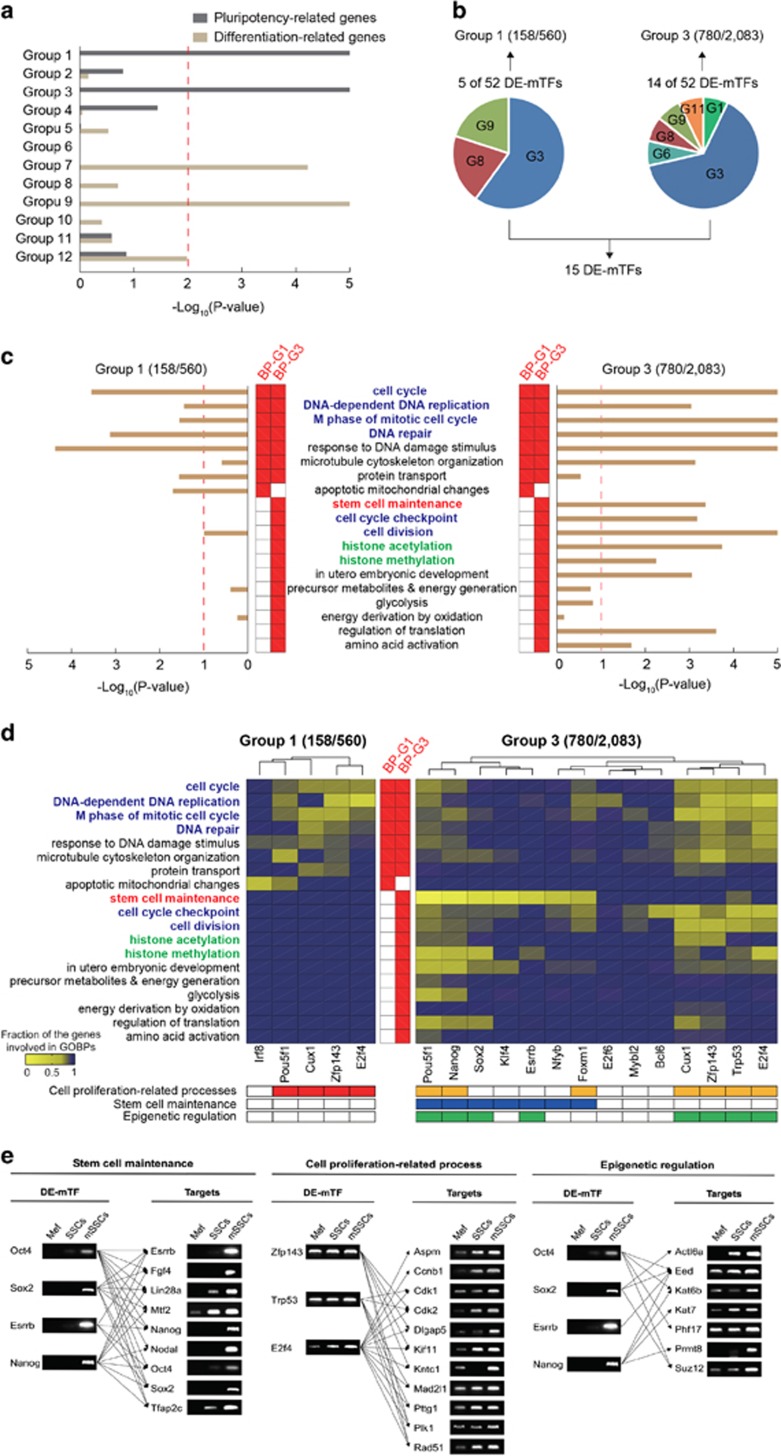
Transcriptional regulatory relationships between DE-mTFs and their target genes. (**a**) Overlaps of groups 1–12 with pluripotency-related genes in mESCs. Groups 1 and 3 significantly (*P*<0.01) overlapped with 844 pluripotency-related genes, whereas groups 7 and 9 significantly (*P*<0.01) overlapped with 1178 differentiation-related genes. The red line denotes the cutoff *P*-value. (**b**) DE-mTFs that significantly (FDR<0.05) regulate the genes in groups 1 and 3. The pie chart shows which groups of DEGs included the five and 14 DE-mTFs for groups 1 and 3, respectively. (**c**) The significance of the GOBPs that are regulated by the 52 DE-mTFs in groups 1–12. The significance for each pair of DE-mTF and GOBP was estimated as the enrichment *P*-value (*x*-axis) obtained from the DAVID software for 158 and 780 target genes in groups 1 and 3, respectively. The default *P*-value cutoff in DAVID is denoted as red dotted lines. The red boxes at the center indicate GOBPs significantly enriched by all the genes in groups 1 (BP-G1) and 3 (BP-G3). (**d**) The significance of the GOBPs that are regulated by the five and 14 DE-mTFs for groups 1 (left) and 3 (right), respectively. The significance for each pair of DE-mTF and GOBP was computed as the fraction of the genes regulated by the DE-mTF among all the genes DEGs involved in the GOBP. The color gradient shows the relative proportions of such genes. The red boxes at the center indicate representative GOBPs in groups 1 (BP-G1) and 3 (BP-G3). The tables show the association of the DE-mTFs with cell proliferation-related processes (red for group 1 and orange for group 3), stem cell maintenance (blue) and epigenetic regulation (green). (**e**) PCR analysis of the differential expression of four representative DE-mTFs and their representative target genes involved in stem cell maintenance and epigenetic regulation as well as three representative DE-mTFs (*Zfp143*, *Trp53* and *E2f4*) and their representative target genes involved in cell proliferation-related processes

Our aim was to understand the TRN that describes the regulation of pluripotency-related genes by the 52 DE-mTFs during the SSC-to-mSSC reprogramming. The GOBP enrichment analysis of groups 1–12 above showed that groups 1 and 3 included the DEGs closely associated with pluripotency (Figure 1c), suggesting that some groups of DEGs are more relevant to pluripotency. To identify the groups of DEGs that are more relevant to pluripotency, we first selected 844 pluripotency-related and 1,178 differentiation-related genes whose expression decreased and increased, respectively, commonly during the differentiation of three different mouse ESC lines (R1, V6.5 and J1) to embryoid bodies (EBs) (Supplementary Figure S1a; Supplementary Table S4) by analyzing the previously reported gene expression profiles (GSE2972, GSE3749 and GSE3231)^19^ (‘Materials and Methods' section). We then compared these genes with groups 1–12 and found that groups 1 and 3 showed the most significant (*P*<10^−5^) overlaps with the 844 pluripotency-related genes, whereas groups 7 and 9 showed the most significant overlaps with the 1178 differentiation-related genes (Figure 3a). This analysis suggests that groups 1 and 3 should be the most relevant to the pluripotency acquired in mSSCs during the SSC-to-mSSC reprogramming, which was consistent with the finding from the GOBP enrichment analysis of groups 1–12 (Figure 1c). Thus, to decode the TRN underlying the pluripotency acquired in mSSCs, we focused on groups 1 and 3, which showed early and late changes during the course of the SSC-to-mSSC reprogramming.

To understand the transcriptional regulatory relationships between the DE-mTFs and their target genes in groups 1 and 3, we then examined how many genes in groups 1 and 3 are regulated by the DE-mTFs. Interestingly, of the 52 DE-mTFs, 5 and 14 (a total of 15 DE-mTFs) significantly (FDR<0.05) regulated the genes in group 1 (158 of 560 genes) and group 3 (780 of 2083 genes), respectively (Figure 3b). Moreover, the target genes of these DE-mTFs in groups 1 and 3 could significantly represent the pluripotency-related cellular processes (Figure 1b) represented by all the genes in groups 1 and 3 (Figure 3c). The 158 target genes in group 1 were associated with cell proliferation-related processes (cell cycle, DNA replication, M phase of mitotic cell cycle, DNA repair) (Figure 3c, left). In addition, the 780 target genes in group 3 were associated with stem cell maintenance, cell proliferation-related processes (cell cycle, DNA replication, M phase of mitotic cell cycle, DNA repair, cell cycle checkpoint and cell division), and epigenetic regulation-related processes (histone acetylation and methylation) (Figure 3c, right).

More specifically, four of the 5 DE-mTFs for group 1 (*Oct4/Pou5f1*, *Cux1*, *Zfp143* and *E2f4*) strongly regulated the target genes in group 1 associated with cell proliferation-related processes (cell cycle, DNA replication, M phase of mitotic cell cycle and DNA repair) (Figure 3d, left). In addition, seven of the 14 DE-mTFs for group 3 (*Oct4/Pou5f1*, *Nanog*, *Foxm1*, *Cux1*, *Zfp143*, *Trp53* and *E2f4*) strongly regulated the genes in group 3 associated with cell proliferation-related processes (cell cycle, DNA replication, M phase of mitotic cell cycle, DNA repair, cell cycle checkpoint and cell division) (Figure 3d, right). Moreover, another seven (*Oct4/Pou5f1*, *Nanog*, *Sox2*, *Klf4*, *Esrrb*, *Nfyb* and *Foxm1*) strongly regulated the genes in group 3 associated with stem cell maintenance, whereas eight (*Oct4/Pou5f1*, *Nanog*, *Sox2*, *Esrrb*, *Cux1*, *Zfp143*, *Trp53* and *E2f4*) regulated the genes in group 3 associated with epigenetic regulation-related processes (histone acetylation and methylation) (Figure 3d, right). These data indicate that the regulation of the target genes in groups 1 and 3 by the 15 DE-mTFs represent the transcriptional regulatory relationships required for the pluripotency acquired in mSSCs during the SSC-to-mSSC reprogramming. A PCR analysis confirmed the differential expression of four representative DE-mTFs (*Oct4/Pou5f1*, *Sox2*, *Esrrb* and *Nanog*) and their representative target genes involved in stem cell maintenance (*Esrrb*, *Fgf4*, *Lin28a*, *Mtf2*, *Nanog*, *Nodal*, *Oct4/Pou5f1*, *Sox2* and *Tfap2c*) and epigenetic regulation (*Actl6a*, *Eed*, *Kat6b*, *Kat7*, *Phf17*, *Prmt8* and *Suz12*), as well as three representative DE-mTFs (Zfp143, Trp53 and E2f4) and their representative target genes involved in cell proliferation-related processes (*Aspm*, *Ccnb1*, *Cdk1*, *Cdk2*, *Dlgap5*, *Kif11*, *Kntc1*, *Mad2l1*, *Pttg1*, *Plk1* and *Rad51*) (Figure 3e).

### Hypothetical TRNs delineating the pluripotency-related processes during the reprogramming of SSCs to mSSCs

**Figure 4:**
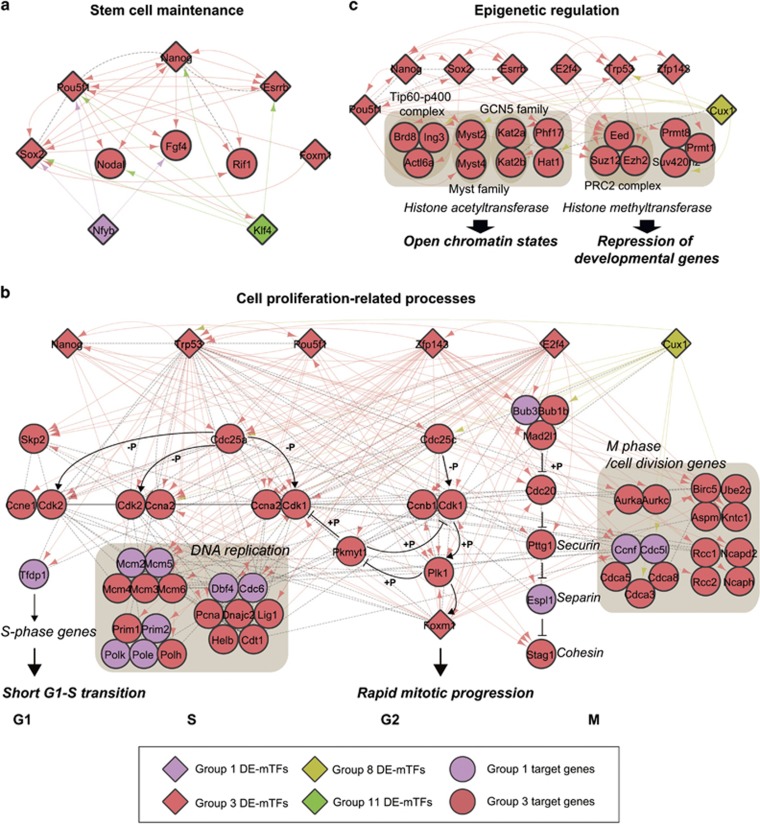
TRNs delineating the pluripotency-related processes during the reprogramming of SSCs to mSSCs. (**a–c**) TRNs underlying the regulation of the genes involved in pluripotency-related processes— stem cell maintenance (**a**), cell proliferation-related processes (**b**) and epigenetic regulation (**c**). The node shapes represent DE-mTFs (diamond) and target genes (circles) and the node colors represent the groups that include the nodes (see node legend box at the bottom). The arrows represent TF-target gene relationships, and the dotted lines represent Protein–protein interactions. The same colors were used for the TFs and the arrow edges. The nodes in the three TRNs were arranged according to the KEGG pathways associated with the three pluripotency-related processes. On the basis of the KEGG pathways, +p and −p represent phosphorylation and dephosphorylation reactions, respectively, and the suppression symbols denote the repression reactions. The background represents a network module that includes the nodes involved in the corresponding cellular process.

We next reconstructed hypothetical TRNs that describe the regulation of the pluripotency-related processes (stem cell maintenance, cell proliferation and epigenetic regulation) by the 15 DE-mTFs, based on the transcriptional regulatory relationships mentioned above between the 15 DE-mTFs and the genes in groups 1 and 3 associated with the pluripotency-related processes. It has been shown that pluripotency-inducing TFs, such as *Oct4/Pou5f1*, *Sox2*, *Myc* and *Klf4*, act as the most essential factors for stem cell maintenance.^11, 12^ In addition to these TFs, recently, a number of regulatory factors, including Nanog, *Lin28* and *Glis1*, have also been reported to be essential for stem cell maintenance.^30, 31^ The TRN for stem cell maintenance showed dense transcriptional regulatory relationships among a set of regulatory factors (Figure 4a), including seven TFs (*Oct4/Pou5f1*, *Sox2*, *Nanog*, *Klf4*, *Esrrb*, *Foxm1* and *Nfyb*), two signaling molecules (*Nodal* and *Fgf4*), and a DNA replication factor (*Rif1*). *Nfyb* in group 1 was up-regulated to positively regulate *Oct4/Pou5f1*, *Sox2* and *Fgf4* in group 3 in the early phase of the SSC-to-mSSC reprogramming, and the regulatory factors in group 3 showed tight regulatory relationships among themselves. Interestingly, the expression of Klf4 decreased during the early phase of the reprogramming and then was restored to positively regulate the regulatory factors in group 3 (*Oct4/Pou5f1*, *Sox2*, *Nodal*, *Nanog* and *Esrrb*) during the late phase of the reprogramming. Altogether, these data suggest the temporal coordination of transcriptional regulation among the regulators associated with stem cell maintenance during the SSC-to-mSSC reprogramming.

Pluripotent stem cells have increased cell cycle activity, reflecting their rapid cell division, which is indicated by a short G1 phase for fast cell cycle progression and enhanced genomic integrity due to fewer accumulated genetic lesions.^32^ The TRN for the cell proliferation (Figure 4b) showed that the genes involved in the following processes associated with fast cell cycle progression were up-regulated during the early phase of the reprogramming (group 1): G1–S transition (*Tfdp1*), DNA replication at S phase (*Mcm2/5*, *Polk/e*, *Prim2*, *Dbf4* and *Cdc6*) and M phase (*Bub3*, *Espl1*, *Ccnf* and *Cdc5I*). Moreover, the genes involved in DNA repair (*Rad54*, *Fancd2*, *Msh6*, *Eef1e1*, *Chaf1a*, *Mgm*, *Gtf2h1*, *Sod2* and *Apex1*) that are associated with enhanced genomic integrity were also up-regulated during the early phase (group 1) (Supplementary Figure S2). These early up-regulated genes are regulated mainly by four DE-mTFs (*E2f4*, *Zfp143*, *Oct4/Pou5f1* and *Cux1*), as shown in Figure 3d. The TRN also showed that a majority of the genes involved in cell cycle progression and genomic integrity were up-regulated during the late phase of the reprogramming (group 3; red nodes in Figure 4b) and were regulated predominantly by seven DE-mTFs (*E2f4*, *Zfp143*, *Oct4/Pou5f1*, *Nanog*, *Trp53*, *Cux1* and *Foxm1*), as shown in Figure 3d. These data demonstrate the temporal coordination of the transcriptional regulation of cell proliferation-related processes by these seven DE-mTFs during the early and late phases of the SSC-to-mSSC reprogramming.

It has been shown that the reprogramming of epigenetic regulation is also important for acquiring pluripotency.^33^ The TRN for epigenetic regulation showed the factors up-regulated during the late phase of the SSC-to-mSSC reprogramming (Figure 4c). These factors included mostly histone acetyltransferases and methyltransferases, to open chromatin states and repress the expression of development-related genes, respectively. Of the histone acetyltransferases, in particular, Tip60–p400 complex genes (*Brd8*, *Ing3* and *Actl6a*) and GCN5 (*Kat2a/b*) and Myst (*Myst2/4*) family genes, as well as *Hat1* and *Phf17*, were up-regulated during the late phase. Of the histone methyltransferases, PRC2 complex genes (*Eed*, *Suz12* and *Ezh2*) and PRMT family genes (*Prmt1/8*), as well as Suv420h2, were up-regulated. The TRN showed that these genes were regulated predominantly by eight DE-mTFs (*Cux1*, *Oct4/Pou5f1*, *Sox2*, *Zfp143*, *Trp53*, *Esrrb*, *Nanog* and *E2f4*), as shown in Figure 3d. Interestingly, of them, Cux1 was down-regulated during the early phase of the reprogramming, whereas the other seven TFs were up-regulated during the late phase. This indicates that the regulation of epigenetic factors by *Cux1* was suppressed, followed by positive regulation of these factors by the other seven TFs, suggesting temporal coordination in the transcriptional regulation of the epigenetic regulation by the eight DE-mTFs during the SSC-to-mSSC reprogramming.

## Discussion

In this study, we attempted to decode the TRN underlying the reprogramming of SSCs to mSSCs through a comparative analysis of gene expression profiles obtained from SSCs, iSSCs and mSSCs and an integrative analysis of (1) DEGs during the SSC reprogramming and (2) TF-target information. These analyses revealed that three key cellular processes (stem cell maintenance, cell proliferation-related processes and epigenetic regulation) were closely associated with the pluripotency acquired during the SSC-to-mSSC reprogramming. These TRNs suggested important features of temporal coordination in the transcriptional regulation of the genes involved in these pluripotency-related processes during the reprogramming. First, four DE-TFs (*E2f4*, *Zfp143*, *Oct4/Pou5f1* and *Cux1*) strongly regulated the genes involved in cell proliferation-related processes during the early transition from SSCs to iSSCs (Figure 4b). Second, seven DE-mTFs (*E2f4*, *Zfp143*, *Oct4/Pou5f1*, *Nanog*, *Trp53*, *Cux1* and *Foxm1*) strongly regulated the genes involved in cell proliferation-related processes, stem cell maintenance and epigenetic regulation (Figures 4a and c).

Our study revealed novel molecular signatures that might be related to pluripotency. For example, the TRN for epigenetic regulation showed that *PRMT1* and *PRMT8*, involved in arginine methylation, are regulated by *ZFP143* and *NANOG*, which regulate the expression of many pluripotency-related genes. This suggested the potential role of arginine methylation in the acquired pluripotency in mSSCs. In addition, the DEGs included several aminoacyl-tRNA synthetases (ARSs). Although ARSs have classically been thought of as housekeeping genes, recent studies have shown that they can have pathophysiological roles in tumorigenesis^21^ and other diseases.^34^ Among the 23 ARSs genes, 19 were up-regulated during the SSC-to-mSSC reprogramming (Supplementary Table S5). Considering that these ARS genes are implicated in cancer cells, which share the ability of rapid cell proliferation with self-renewing stem cells,^35, 36, 37, 38^ the changes in expression of the ARS genes during the SSC-to-mSSC reprogramming may indicate their potential roles in the acquired pluripotency in mSSCs.

Rapidly dividing pluripotent stem cells have different cellular metabolic regulation processes compared with their differentiated cells. In particular, pluripotent stem cells show increased levels of glycolysis under aerobic conditions, leading to high glycolytic fluxes to (1) generate ATP and intermediate metabolites through the pentose phosphate pathway for lipid and nucleotide biosynthesis and (2) reduce the levels of reactive oxygen species (ROS) that can cause stem cell differentiation.^39^ To examine this, we also reconstructed a hypothetical TRN that delineated the regulation of the genes involved in aerobic glycolysis (Supplementary Figure S3). The TRN contained seven genes (*Lin28a*, *Pfkp*, *Aldoa*, *Pgam1*, *Eno1*, *Pdhb* and *Pdk1*) involved in glycolysis, which were up-regulated during the late phase of the SSC-to-mSSC reprogramming. The expression changes of these genes are collectively regulated by Oct4/Pou5f1 and Nanog during the late phase. In addition, two genes (*Sod2* and *Gpx1*) involved in the reduction of ROS levels are mainly regulated by Nanog and Trp53. These TFs (*Pou5f1*, *Nanog* and *Trp53*) also regulate the cell cycle-related genes that are up-regulated during the early and late reprogramming stages (Figure 4a). These data indicate temporal coordination in the transcriptional regulation of the cell cycle and aerobic glycolysis by these TFs.

Owing to the incompleteness of the TF-target interactome, our TRNs provide only a partial view of the transcriptional regulation activated during the SSC-to-mSSC reprogramming. Accordingly, the 15 DE-mTFs on which we focused for understanding the transcriptional regulation during the reprogramming could be incomplete. In our study, we identified the 52 DE-mTFs that can contribute to the reprogramming. ChIP-chip or ChIP-seq analysis of these DE-mTFs could provide more complete views of the transcriptional regulation underlying the temporal expression changes of the genes associated with the SSC-to-mSSC reprogramming. Despite the limitations of the TF-target interactome, however, the 15 DE-mTFs and the TRNs still provide meaningful insights into the transcriptional regulatory mechanisms for the early and late activation of cellular processes during reprogramming, as well as the temporal coordination of the transcriptional regulatory mechanisms as demonstrated in Figures 4a–c.

Our analysis revealed that five DE-mTFs (*E2f4*, *Zfp143*, *Oct4/Pou5f1*, *Irf8* and *Cux1*) strongly regulated the genes in group 1, which were up-regulated during the early phase of the SSC-to-mSSC reprogramming. Of the five DE-mTFs, Cux1 was down-regulated during the early phase of the reprogramming, but the other four were up-regulated (*E2F4*, *Zfp143* and *Oct4/Pou5f1*) or down-regulated (*Irf8*) during the late phase. However, it is not clear how the three late up-regulated TFs (*E2F4*, *Zfp143* and *Oct4/Pou5f1*) can regulate the early up-regulated genes in group 1. The DEGs were identified based on the discrete decision of whether a gene showed a statistically significant change in the three comparisons (iSSCs versus SSCs, mSSCs versus SSCs and mSSCs versus iSSCs). This discrete decision may not be able to detect meaningful marginal expression changes of the DE-mTFs that can contribute to induction of the genes up-regulated during the early phase of the SSC-to-mSSC program. However, the late up-regulated DE-mTFs can also modulate the expression of the early up-regulated genes during the late phase of the reprogramming. This modulation can significantly contribute to the stabilization of the up-regulation of the early responsive genes during the late phase of the reprogramming.

Our network model (Figure 4c) for epigenetic regulation suggests that eight DE-mTFs (*Cux1*, *Oct4/Pou5f1*, *Sox2*, *Zfp143*, *Trp53*, *Esrrb*, *Nanog* and *E2f4*) can regulate the induction of histone methyltransferases (*Eed*, *Suz12* and *Ezh2* in the PRC2 complex and *Prmt1*, *Prmt8* and *Suv420h2*) during the SSC-to-mSSC reprogramming. This is consistent with a previous finding that the reprogramming of epigenetic regulation is also important for acquiring pluripotency.^33^ Interestingly, of these methyltransferases, *Prmt1* and *Prmt8* are arginine methyltransferases, and their induction during the SSC-to-mSSC reprogramming thus further suggests the potential association of histone arginine methylation with the pluripotency acquired by mSSCs. Consistent with this observation, Torres-Padilla *et al.*^40^ showed that four-cell blastomeres with reduced developmental potential had lower levels of histone H3 arginine 17 and 26 methylation, whereas cells with higher levels of such methylation were predisposed to contribute to the pluripotent inner cell mass (ICM). They further showed that the increased expression of coactivator-associated-protein-arginine-methyltransferase 1 (*Carm1*) in individual blastomeres led to increased histone methylation, resulting in a higher expression of pluripotency TFs, such as *Nanog* and *Sox2*, and the direction of progeny to the ICM. Altogether with these data, our network model suggests that histone arginine methylation by PRMT1 and PRMT8 may have an important role in acquiring pluripotency during the SSC-to-mSSC reprogramming, as the *Carm1*-dependent arginine methylation was found to be essential in the regulation of pluripotency in the early mouse embryo.

The temporal coordination of the transcriptional regulation suggested by our TRNs can extend the current knowledge of the regulation underlying the SSC-to-mSSC reprogramming. The current understanding of the reprogramming-related regulation mainly focuses on the roles of individual TFs that contribute to the reprogramming. Our study extends this view into the collective transcriptional regulation involving multiple TFs and the dynamic coordination of their transcriptional regulation. To develop more accurate models of the temporal coordination of the transcriptional regulation, comprehensive time-course global assays involving more time points during the transition of iSSCs to mSSCs should be employed and could provide detailed differences in the DE-mTFs and their target genes up-regulated during the late phase of the SSC-to-mSSC reprogramming. In conclusion, our analysis provides key DE-mTFs and TRNs that can be used as the bases for understanding the serial, coordinated activation of cellular processes during the SSC-to-mSSC reprogramming.
