# Supplementary figures and images for: Transcriptional regulatory networks underlying the reprogramming of spermatogonial stem cells to multipotent stem cells
Source: Exp Mol Med. 2017 Apr 14;49(4):e315–. doi: 10.1038/emm.2017.2 (PMC5420799; doi:10.1038/emm.2017.2)

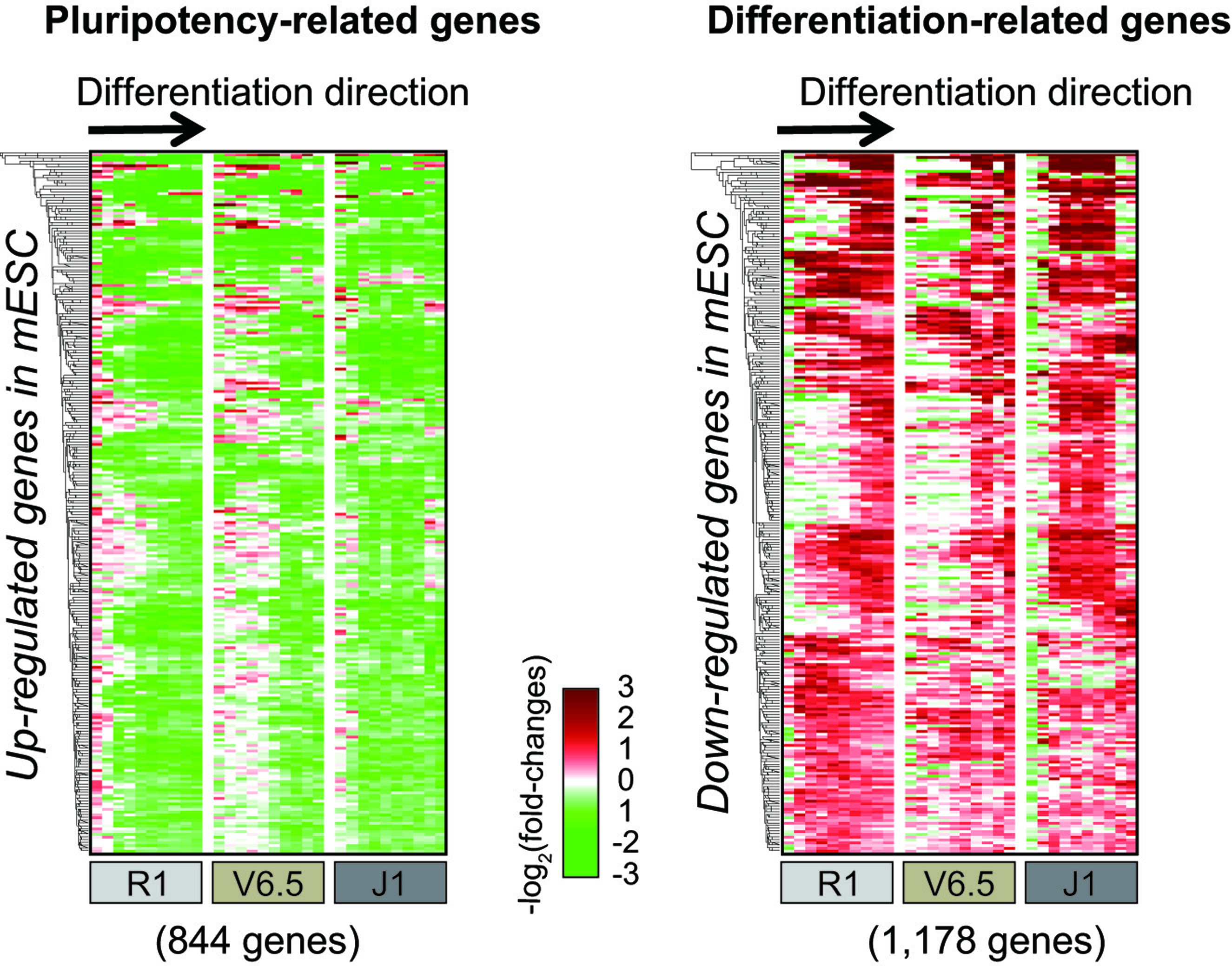

Supplement: Supplementary Figure 1 [file emm20172x1.tif]

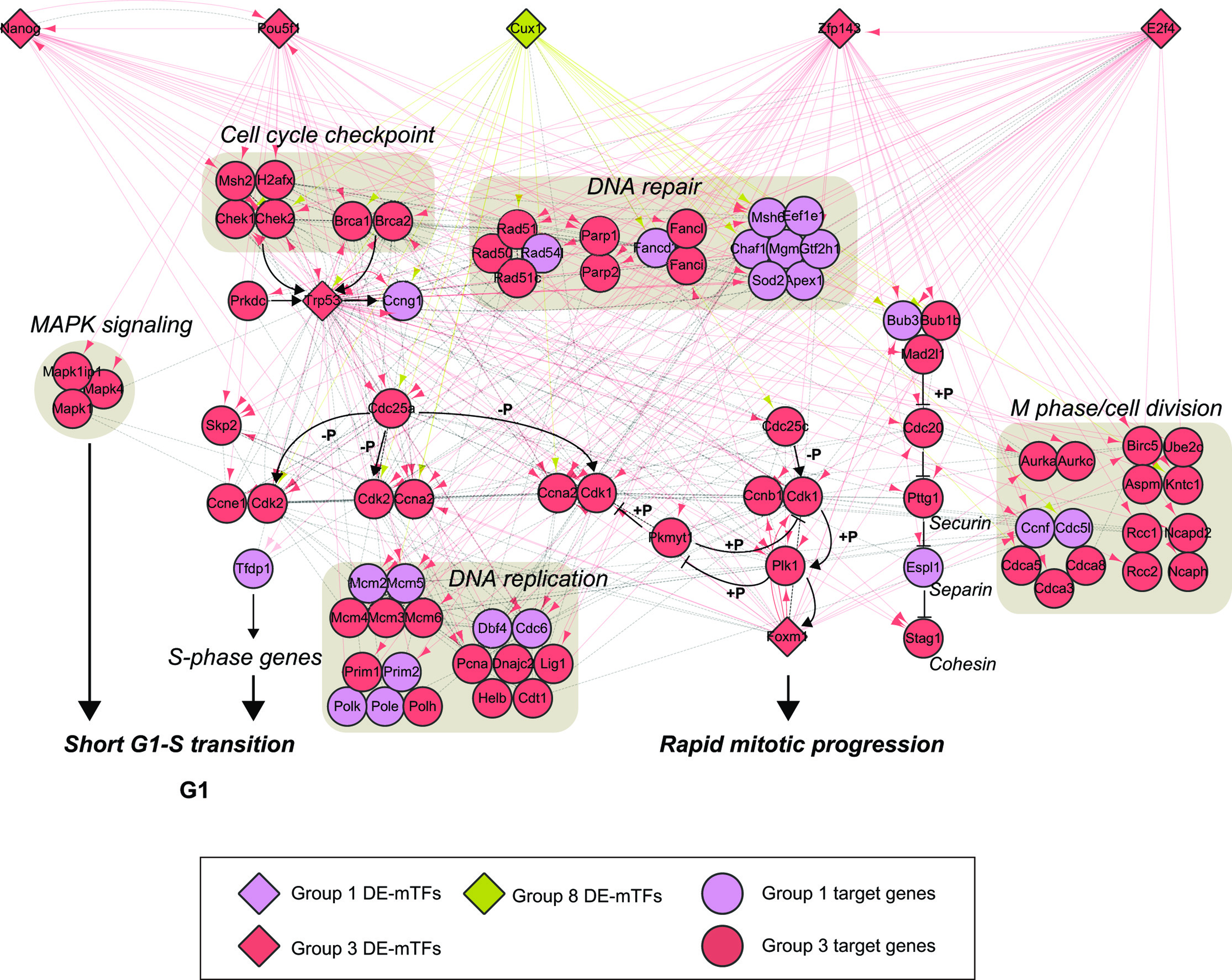

Supplement: Supplementary Figure 2 [file emm20172x2.tif]

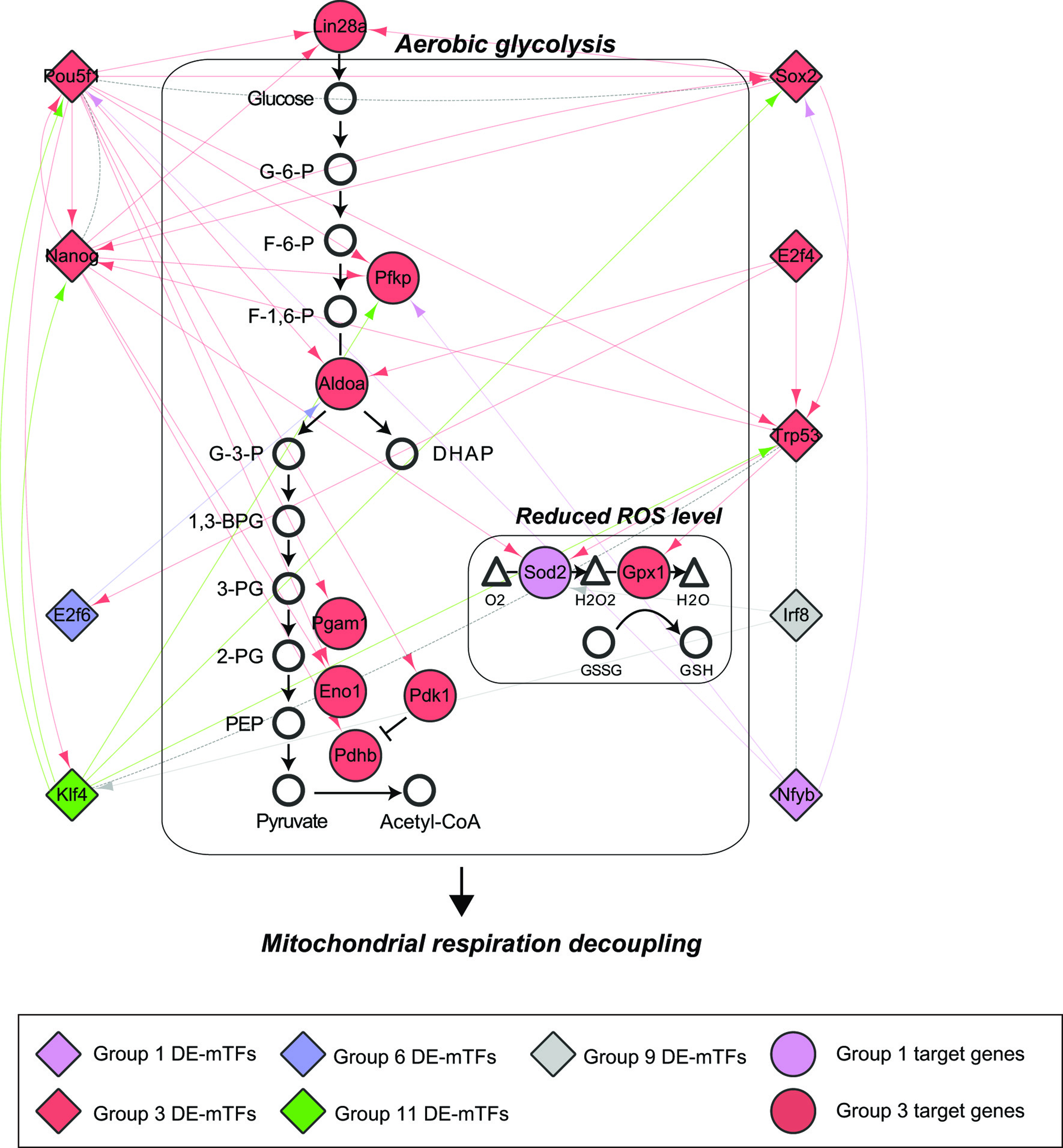

Supplement: Supplementary Figure 3 [file emm20172x3.tif]
